# How do systematic reviews incorporate risk of bias assessments into the synthesis of evidence? A methodological study

**DOI:** 10.1136/jech-2014-204711

**Published:** 2014-12-06

**Authors:** Srinivasa Vittal Katikireddi, Matt Egan, Mark Petticrew

**Affiliations:** 1 Evaluation of Social Interventions Programme, MRC/CSO Social and Public Health Sciences Unit, University of Glasgow, Glasgow, UK; 2 Social and Environmental Health Research Department, London School of Hygiene and Tropical Medicine, London, UK

**Keywords:** SYSTEMATIC REVIEWS, EPIDEMIOLOGY, PUBLIC HEALTH, Epidemiological methods

## Abstract

**Background:**

Systematic reviews (SRs) are expected to critically appraise included studies and privilege those at lowest risk of bias (RoB) in the synthesis. This study examines if and how critical appraisals inform the synthesis and interpretation of evidence in SRs.

**Methods:**

All SRs published in March–May 2012 in 14 high-ranked medical journals and a sample from the Cochrane library were systematically assessed by two reviewers to determine if and how: critical appraisal was conducted; RoB was summarised at study, domain and review levels; and RoB appraisals informed the synthesis process.

**Results:**

Of the 59 SRs studied, all except six (90%) conducted a critical appraisal of the included studies, with most using or adapting existing tools. Almost half of the SRs reported critical appraisal in a manner that did not allow readers to determine which studies included in a review were most robust. RoB assessments were not incorporated into synthesis in one-third (20) of the SRs, with their consideration more likely when reviews focused on randomised controlled trials. Common methods for incorporating critical appraisals into the synthesis process were sensitivity analysis, narrative discussion and exclusion of studies at high RoB. Nearly half of the reviews which investigated multiple outcomes and carried out study-level RoB summaries did not consider the potential for RoB to vary across outcomes.

**Conclusions:**

The conclusions of the SRs, published in major journals, are frequently uninformed by the critical appraisal process, even when conducted. This may be particularly problematic for SRs of public health topics that often draw on diverse study designs.

## Introduction

**Figure 1:**
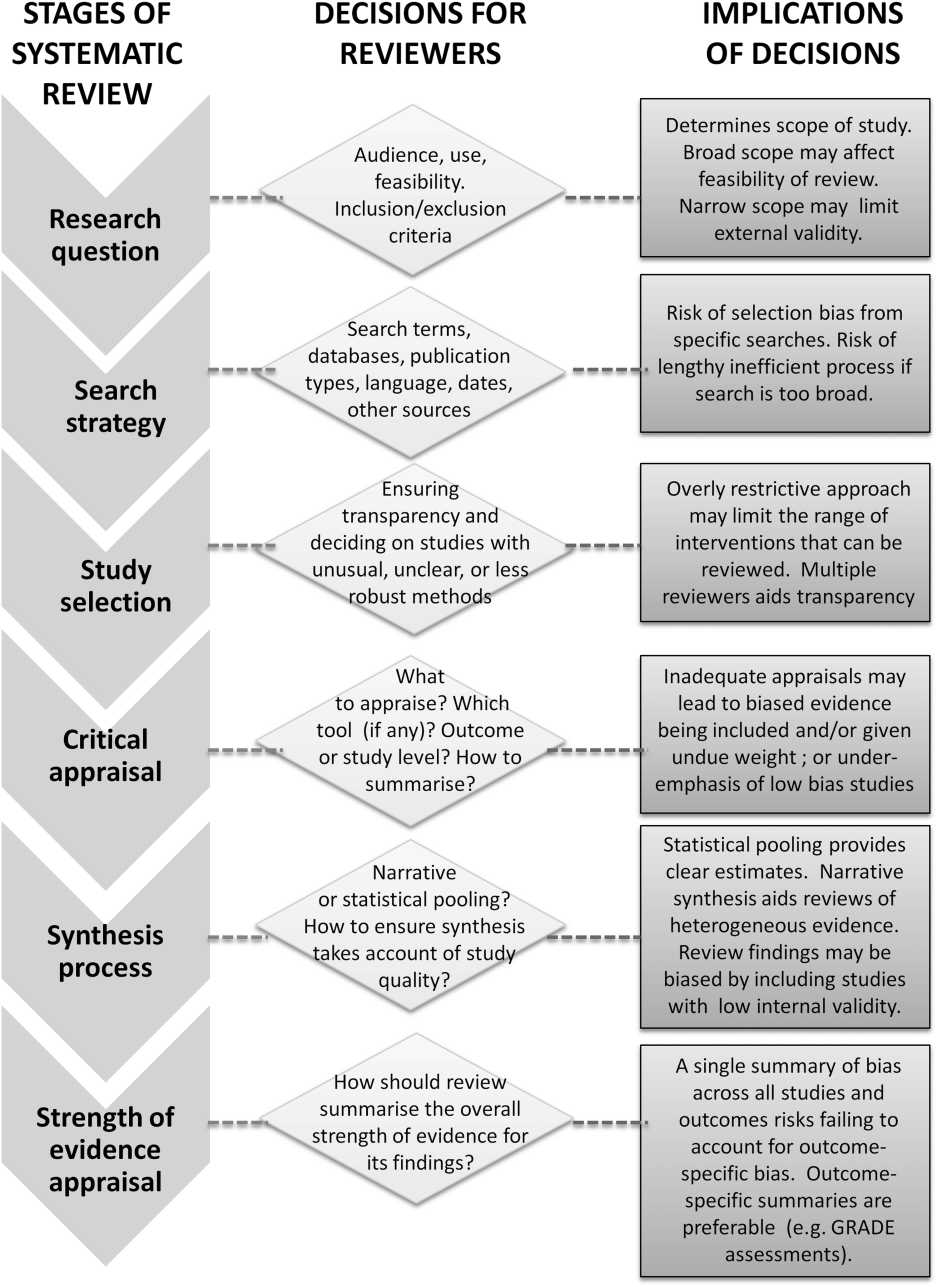
Conceptual model outlining factors to consider when undertaking a systematic review. GRADE, Grading of Recommendations Assessment, Development and Evaluation.

Systematic reviews (SRs) are often considered a ‘gold standard’ form of evidence and inform decision-making across and beyond the health sciences.^1–3^ SRs vary in methods and scope but frequently use a predefined comprehensive search strategy to identify all potentially relevant studies; predefined inclusion criteria to minimise bias arising from the selective consideration of evidence; and assess the risk of bias (RoB) of included studies.^2^ ^4^ Underpinning each stage is a desire to reduce bias by prioritising evidence from the most scientifically valid studies in a transparent and replicable way (see figure 1 for a conceptual model summarising the conduct of SRs).

The term ‘study quality’ is widely used but remains ill-defined, and suggests investigating the extent to which research is conducted to the highest possible standards.^4^ This paper focuses on the narrower concept of RoB, relating exclusively to internal validity. Studies with greater RoB often (but not always) overestimate treatment effect sizes.^5–7^ The findings of SRs that combine low RoB primary studies with those at greater RoB may result in inappropriate recommendations for practice or policy.^2^ ^4^ ^8–10^ To prevent this, critical appraisal is recommended to identify which findings should be emphasised as most reliable.^4^ It aims to move synthesis beyond discredited practices such as ‘cherry picking’, whereby findings that best fit the reviewers’ own interests are emphasised, and ‘vote counting’ in which the reviewers prioritise findings that a majority of studies agree on without considering whether those studies are most robust. Critical appraisal allows reviewers to explore whether contradictory findings between studies reflect differing risks of bias, and so, for example, help establish if findings from a minority of included studies are more valid than the methodologically weaker majority. They can also help reviewers assess whether the overall evidence base for a particular outcome is weak or strong. For example, the Grading of Recommendations Assessment, Development and Evaluation (GRADE) approach to assessing strength of review evidence considers critical appraisals when developing evidence-informed recommendations.^11^ ^12^

To ensure that SRs take account of RoB, it is not enough to simply assess methodological characteristics, nor even to describe those characteristics in a table or text.^4^ ^13^ Reviewers must use their critical appraisals to inform subsequent review stages, notably the synthesis and the conclusion-drawing stages.

Numerous critical appraisal tools are available.^4^ ^14–16^ Tools historically allowed summary scores to be calculated for each randomised controlled trial (RCT) included in an SR.^17^ A summary score is a simple way of identifying high-quality and low-quality studies and also an easy way to incorporate into synthesis. However, some experts view this approach as unsatisfactory since individual components of summary scores have been found to discriminate between studies at high and low RoB to a greater extent than overall scores.^18^ ^19^ Public health often draws upon diverse forms of evidence that extend beyond RCTs, making it even more challenging to identify which studies have the lowest RoB.^14^ ^20^ A further consideration is that RoB may differ across outcomes within the same study.^6^ For example, patient and investigator blinding to outcome assessment may be less important for all-cause mortality than for more subjective measures like pain.

Current Cochrane Collaboration guidance emphasises a move away from rigid checklists and scores to an approach which focuses on methodological ‘domains’ (such as allocation, blinding, completeness of data) that should be tailored on the basis of the review topic. This domain-level approach, which was developed between 2005 and 2007, is intended to have some flexibility and involves subjective judgements made by reviewers regarding, for example, the most appropriate domains to appraise, the relative importance of each domain, and the overall RoB, both within and across studies.^4^ ^21^ These judgements should be outcome-specific and ideally informed by empirical evidence of bias, the likely direction of bias and the likely magnitude of bias. However, this may be difficult to achieve in practice, given the acknowledged evidence gap in the relative importance of different domains of bias.^4^

Incorporation of RoB assessments into synthesis is crucial to ensure that SR conclusions are based on the best available evidence. Failure has serious implications for evidence-informed policy and practice. In this study, we explore if and how critical appraisals inform the synthesis and interpretation of evidence in recent SRs published in high-impact journals and in the Cochrane database.

## Methods

SRs published in 14 journals, between March 2012 and May 2012 inclusive, were identified by manual searches carried out independently by two reviewers (SVK and ME). These journals were purposively chosen to provide coverage of highly ranked journals within the fields of general medicine, general practice, paediatrics and public health (see web appendix or table 1 for list of journals). To be included in the study, the reviews had to be published in journal issues during this period (and not in online first format only) and self-defined by the authors as SRs. The first five new Cochrane SRs published in each of these months were also considered, as Cochrane SRs have been reported as being conducted to a higher methodological quality than those published elsewhere.^22^ ^23^ When SRs do not attempt to synthesise findings across studies (eg, those conducted to study review methodology), assessments of RoB do not usually inform the synthesis process and were therefore excluded. Additional details were retrieved if provided in appendices and published protocols, or cited in separate documents.

A standard data extraction template was created in Microsoft Excel 2007 based on guidelines produced by the Cochrane Collaboration^4^ (see online appendix). These included details about the study characteristics, the critical appraisal process, the evidence synthesis approach taken and how critical appraisal informed the findings presented in the SR. Data extraction included abstractions based on predefined categories as well as qualitative text data (to allow a combination of systematic assessment and depth to be achieved). Both reviewers carried out data extraction independently, with disagreements resolved by consensus and discussion with a third author (MP).

The categories for coding data extraction were as follows: Studies were grouped by topics into ‘healthcare intervention’ (intervention delivery within a health services setting); ‘other health intervention’ (intervention study but delivered in a non-health services setting); ‘observational epidemiology’ and ‘qualitative’ study. The type of critical appraisal tool was categorised under: ‘standard tool’ when authors used a previously published tool (eg, Cochrane risk of bias tool, Hamilton tool) without modification; ‘adapted tool’ when a previously published tool was modified by the authors for their review purpose; ‘bespoke tool’ when a new tool was created by the authors; ‘description’ when information from included studies was extracted but not related to RoB (including data extraction using reporting guidelines, such as the STROBE statement); and ‘none’ when no information relevant to RoB was extracted. Further information collected about critical appraisal included whether a separate appraisal was conducted for each outcome and if the individual criteria used in the critical appraisal were reported. Where further information on the critical appraisal tool was provided in a protocol or citation, the original material was retrieved.

We determined if the reviews allowed readers to rank studies by RoB, and for those that did, how: use of a ‘summary score’ (where the number of RoB criteria were added together); ‘threshold summary score’ (where a summary score was deemed low RoB, if achieving a cut-off score); ‘weighted score’ (where criteria are added together, with some factors given greater weighting); ‘specific domains prioritised’ (when some RoB domains were considered most important but without utilising a numeric score); ‘all criteria required’ (when all of the appraisal criteria needed to be met for the study to be deemed low RoB); ‘unclear’ (when it was difficult to determine the process by which authors differentiated studies by their RoB). Information on whether RoB assessments were incorporated was assessed by reviewing all available published information, with even minimal evidence for incorporation acceptable. In addition, how incorporation of RoB was achieved in the synthesis (sensitivity analysis, narrative discussion, exclusion of studies at high RoB and ‘other’) was also extracted. Lastly, the levels at which RoB was summarised in reviews was noted. This included determining whether RoB was assessed at the domain level within studies included in a review, at the study level, across studies, and at the review level (including how).

## Results

A total of 59 SRs that met the inclusion criteria were identified (table 1). Considerable diversity was achieved in the sample of SRs, with reviews considering a broad range of research questions and synthesising a wide variety of types of evidence (see web appendix table 1 for further details of each included review). Most reviews were either focused on evaluating healthcare interventions (42%) or observational epidemiology (37%) with other types of health intervention (such as preventive interventions or clinical management tools) and qualitative reviews comprising a minority.

**Table 1:** Characteristics of systematic reviews included in analysis

	Number of reviews	% of category total
Topic of systematic review		
Healthcare intervention	25	42
Other health intervention	9	15
Observational epidemiology	22	37
Qualitative	3	5
Journal		
Addiction	2	3
American Journal of Public Health	2	3
Annals of Family Medicine	3	5
Annals of Internal Medicine	8	14
BMJ	8	14
British Journal of General Practice	2	3
Cochrane Database	14	24
JAMA	3	5
Lancet	3	5
Pediatrics	6	10
PLoS Medicine	3	5
Preventive Medicine	1	2
Social Science and Medicine	4	7
Number of outcomes		
Single	11	19
Multiple	48	81
Summative synthesis		
Meta-analysis	44	75
No meta-analysis	15	25

All except 6 (10%) of the reviews conducted critical appraisal as part of the review process (table 2). In most cases, this involved the use or adaptation of an existing critical appraisal tool. However, two studies used guidelines for study reporting (such as STROBE^24^) rather than tools for appraising RoB. Of the 42 studies that investigated multiple outcomes through a critical appraisal, 15 did not carry out separate critical appraisals for each outcome.

**Table 2:** Details regarding critical appraisal used in systematic reviews

	Numbers of reviews	% of category total
Critical appraisal		
Standard tool (pre-existing tool used without modification)	37	63
Adapted tool (pre-existing tool adapted for review)	10	17
Bespoke tool (new tool created by authors)	4	7
Description (reporting of study characteristics only)	2	3
Other	0	0
None	6	10
Separate appraisal per outcome		
Yes	27	52
No	15	29
N/A (single outcome review)	10	19
Domain-level assessments of risk of bias (eg, outcomes blinded, selective outcome data)		
Domain-level risk of bias presented	25	48
Individual criteria grouped into domains presented	5	10
No domain summary for risk of bias	22	42
Individual appraisal criteria reported		
Yes	26	50
No	26	50
Critical appraisal allows ranking of studies		
Yes	28	54
No	24	46

Twenty eight (54%) of the SRs ranked studies by RoB, or at least provided sufficient information to enable readers to differentiate between which studies were at higher RoB and which studies were at lower RoB. Of these, eight reviews used an approach based on summary scores of criteria to identify those studies at the lowest RoB (table 3). In five cases, all criteria were required for a study to be considered at low RoB and only a further five SRs adopted the Cochrane Handbook's approach of prioritising specific domains to determine which studies had the lowest RoB. Of the four Cochrane reviews to allow included studies to be ranked by RoB, three explained which domain assessments formed the basis for prioritisation. However, none of the reviews, including the Cochrane reviews, provided a justification for why these domains had been prioritised; and in six reviews, it was unclear on what basis the studies had been identified as being at a high or low RoB.

**Table 3:** Methods for ranking included studies by risk of bias in systematic reviews

	Numbers of reviews	% of category total
Simple summary score (criteria met added together)	4	14
Cut-off threshold score (summary score dichotomised on the basis of a cut-off)	4	14
Weighted score (criteria added together, with some factors given greater weighting as deemed more important)	1	4
Specific domains prioritised (some risk of bias domains, such as allocation concealment or blinding, deemed more important)	5	18
All criteria required	5	18
Unclear	6	21
Only one criterion difference between included studies	1	4
Combination of score and domain prioritisation	2	7

In 20 reviews, critical appraisal did not explicitly inform the synthesis stage, and therefore did not appear to influence the review findings (table 4). SRs that only included RCTs to study intervention effectiveness more commonly incorporated RoB (17 of 24, 71%), compared to reviews that included RCTs alongside other designs (8 of 12, 67%) and those that only included other designs (10 of 23, 57%). For reviews that made use of RoB assessments, these were most commonly incorporated into the synthesis process, either narratively or through sensitivity analysis. Of the six Cochrane reviews that planned to carry out sensitivity analysis, half of them were unable to do so due to the small number of included studies, with the result that two reviews of RCTs appeared to neglect quality in the synthesis process. A small number of studies (n=6) incorporated RoB assessments into synthesis using multiple approaches. These assessments were used in a variety of other ways, as shown in table 4 and the web appendix. When RoB was not incorporated into synthesis, examples of vote counting were found,^25–27^ even though the study methods had been assessed.

**Table 4:** Methods for Incorporating risk of bias assessments into reviews during synthesis

	Number of reviews	% of category total
Were risk of bias assessments incorporated into synthesis?		
Yes	37	63
No	20	34
Not applicable	2	3
How were risk of bias assessments incorporated into synthesis?*		
Sensitivity analysis (eg, limiting to studies at lowest risk of bias in a secondary analysis)	20	54
Narrative (discussion within text)	14	38
Exclusion of studies at high risk of bias from main review synthesis	5	14
Other approach	4	11

*Denominator is the number of reviews that incorporated risk of bias into the synthesis process. Note that the total adds up to more than 100% because some reviews used multiple methods. The list of other approaches used is available in the web appendix.

SRs assessing multiple outcomes frequently (n=12) summarised RoB at the study level across outcomes, thereby assigning the same RoB assessment to a study irrespective of the potential for bias for each outcome (table 5). One-third of the studies summarised bias at the review level. In most cases, these review-level summaries of bias used standardised approaches such as the GRADE guidelines.

**Table 5:** Summaries of risk of bias conducted by systematic reviews

	Number of reviews	% of category total
Was a summary of risk of bias presented at the study level?		
No risk of bias assessment at the study level	26	45
Review studying a single outcome which presents risk of bias at the study level	7	12
Review studying multiple outcomes which summarise the risk of bias at the study level but does not assess the risk of bias separately for each outcome	12	21
Review studying multiple outcomes which summarise the risk of bias separately for each outcome	13	22
Was a summary of the risk of bias presented for each outcome across studies?		
Yes	30	52
No	28	48
Was a review-level summary risk of bias provided?		
Yes	19	33
No	39	67
How was the risk of bias summarised at the review level?*		
GRADE	11	58
Cochrane risk of bias table	7	37
Narrative statement (in text)	4	21

*Denominator is the number of reviews providing a review-level summary risk of bias. Note that the total adds up to more than 100% because some reviews used multiple methods for summarising bias at the review level.

GRADE, Grading of Recommendations Assessment, Development and Evaluation.

## Discussion

Although critical appraisals of the included studies are frequently conducted in SRs published in major journals, the conclusions of those reviews are frequently uninformed by this process. SRs that focus on study designs other than RCTs may be particularly subject to this problem. There have been instances of reviewers not carrying out critical appraisals. Assessing RoB using scoring systems continues, despite the Cochrane Collaboration's recommendations to avoid their use. More strikingly, the practice of carrying out a critical appraisal which does not subsequently inform findings of the synthesis process appears common. This resulted in some reviews engaging in ‘vote counting’, where the number of studies is counted to provide an indication of the strength of evidence. Ignoring critical appraisal in this way may result in policy and practice recommendations not based on the best available evidence, thereby threatening the validity of the SR process. Approaches to incorporating RoB assessments into the findings of SRs are (arguably appropriately) varied but frequently lack transparency. Lastly, some SRs that investigate multiple outcomes continue to ignore the potential for RoB to differ across outcomes.

Our study has a number of strengths. We adopted a structured approach to investigate SR practices, making use of two independent reviewers. Our methods allowed detailed investigations of how RoB is assessed and then incorporated the assessments into the findings of SRs. However, some limitations are noteworthy. First, the SRs examined are not a random sample of all works that had been published. Instead, we assessed SR practice by analysing reviews published in high-quality journals across a number of areas of health research. Our study therefore highlights the existence of problematic practices, but most likely underestimates their frequency. Similarly, we have not analysed a sample large enough to provide accurate statistical estimates of the frequency of these practices but instead sought in-depth data obtained from a more qualitative approach. This has allowed us to present a diversity of approaches that are currently being used. Our study is based on a sample of published material only and some practices may not be evident. For example, reviewers not reporting numerically summarised RoB scores may have nevertheless informally calculated them to assist with synthesis. Lastly, the Cochrane guidelines are regularly revised to incorporate developments in best practice and new research.^21^ Therefore, it may be inappropriate to expect all Cochrane reviews to incorporate the latest guidance. However, our findings show that many Cochrane SRs selectively followed some but not all of the recently published guidance.

Much of the previous literature focused on limitations of critical appraisal tools,^15^ ^28^ ^29^ particularly for observational studies,^14^ rather than how RoB assessments are subsequently incorporated into SR findings. Moja *et al*^13^ found that SRs published in 1995–2002 frequently ignored critical appraisals during synthesis, but at the time noted that the methods for assessing and incorporating RoB assessments were in their infancy. Similarly, de Craen *et al*^30^ investigated how SRs of RCTs published in 2002–2003 incorporated RoB assessments into their synthesis. Half of the reviews published in the sample from the Cochrane library and leading general medical journals did not incorporate findings of critical appraisal into their review. Most recently, Hopewell *et al*^31^ reported a lack of RoB incorporation into synthesis and meta-analysis within Cochrane and non-Cochrane SRs of RCTs, with the latter performing more poorly. Although there is evidence that many aspects of SR conduct are improving,^23^ our research demonstrates that problems with the utilisation of critical appraisal in synthesis still persist. Importantly, we document the relevance of this issue for diverse forms of evidence beyond RCTs. By comparing SRs that include RCTs with SRs of observational studies, our findings highlight the particular need for further research on the latter.

Over the past decade, considerable progress has been made in developing guidelines for conducting SRs^4^ ^32^ and developing clinical and public health guidelines.^11^ However, these developments have made the SR process more complicated. Concerns over using RoB summary scores resulted in the Cochrane guidelines arguing for a move away from standardised scoring systems, which combined ease of use with transparency. Our findings not only show that RoB summary scores are still frequently used but also suggest that there is confusion about how best to incorporate critical appraisals into SR findings. This resulted in critical appraisals being ignored, despite having been conducted. Even when appraisals inform SR findings, it is frequently unclear as to how they have been used and the reasons for privileging some studies over others. This lack of clarity threatens the transparency and reproducibility of SRs.

While it is not always appropriate to carry out an SR that meets all the requirements of a Cochrane review,^33^ considering RoB remains important for all reviewers. An overarching principle that may be helpful to remember when conducting synthesis in SRs is to consider what the best available evidence recommends, which may not necessarily reflect the overall evidence base.^20^ At a minimum, this suggests that reviewers should clearly report findings from the most robust studies, either as a sensitivity analysis or in the primary analysis. Depending on the studies being reviewed, statistical and/or narrative techniques may be appropriate. Given that the study design and type of intervention are closely related, reviewers should ideally go further and consider whether an ‘intervention selection bias’ is inadvertently introduced by focusing only on higher quality studies. This means it may be helpful to examine whether the types of interventions evaluated in higher RoB studies differ systematically from lower RoB studies, for example, the latter may focus on individual-level interventions evaluated using RCTs while higher RoB studies may be more likely to include observational evaluations of population-level interventions (eg, public policies).^20^ ^34^

Early tools for critical appraisal were appealing because they were simple to use and resulted in a score which allowed ranking of studies by RoB, facilitating incorporation into the synthesis process.^17^ ^19^ ^35^ Unfortunately, this simplicity came to be regarded as a source of weakness, as well as a strength, and these tools have been replaced by more complex guidance intended to address some of their limitations.^4^ ^21^ ^36^ We recognise the need for this development but take the view, supported we believe by this study, that reviewers are struggling to understand and/or operationalise current guidance on how to conduct and incorporate critical appraisal within synthesis. Further research is required to establish the relative importance of different forms of bias and their likely impact^6^ ^37^ and also to clarify how critical appraisals should be incorporated into SR findings.^38^ ^39^ However, to ensure that SRs really do direct decision-makers to the best available evidence, there is an urgent need to make guidance more understandable to the diverse reviewers involved.

**What is already known?**

- Systematic reviews are a key mechanism for facilitating evidence-informed decision-making and commonly draw upon diverse study designs.
- Critical appraisal is necessary to identify which studies have the lowest risk of bias and is now more consistently conducted within systematic reviews.

**What does this study add?**

- Even when critical appraisal is carried out, it often does not inform the evidence synthesis process, particularly for systematic reviews of non-randomised studies.
- Common methods for incorporating risk of bias assessments into the synthesis process include sensitivity analysis, narrative assessment and restricting the synthesis to studies at a lower risk of bias.
- There is an urgent need for greater clarity in systematic review guidance and understanding among authors that the critical appraisal process must inform the final synthesis; so systematic reviews are based on the best available evidence.

## Supplementary Material

Web supplement
