# Supplementary material for: How do systematic reviews incorporate risk of bias assessments into the synthesis of evidence? A methodological study
Source: J Epidemiol Community Health. 2014 Dec 6;69(2):189–95. doi: 10.1136/jech-2014-204711 (PMC4316857; doi:10.1136/jech-2014-204711)
Supplement: Web supplement [file jech-2014-204711-s1.pdf]

Appendix Table 1 – Detailed Description of Included Systematic Reviews

Publication	First Author	Broad Discipline	Objective	Type of included studies	No. Studies Included	Single/ Multiple outcomes	Meta-analysis
Lancet	Herbert [1]	Observational Epidemiology	To synthesise current evidence on the prevalence of poor diet, inadequate physical activity, and overweight and obesity in prisoners.	Observational Studies	31	Multiple	No
BMJ	Prochaska [2]	Health Technology Intervention	To examine the risk of treatment emergent, cardiovascular serious adverse events associated with varenicline use for tobacco cessation.	RCTs	22	Single	Yes
BMJ	Thangaratinam [3]	Other Health Intervention	To evaluate the effects of dietary and lifestyle interventions in pregnancy on maternal and fetal weight and to quantify the effects of these interventions on obstetric outcomes.	RCTs	44	Multiple	Yes
BMJ	Ker [4]	Health Technology Intervention	To assess the effect of tranexamic acid on blood transfusion, thromboembolic events, and mortality in surgical patients.	RCTs	129	Multiple	Yes
JAMA	Hempel [5]	Health Technology Intervention	To evaluate the evidence for probiotic use in the prevention and treatment of antibiotic-associated diarrhea (AAD).	RCTs	82	Single	Yes

JAMA	Chico [6]	Observational Epidemiology	To conduct a systematic review and meta-analysis of malaria and STI/RTI prevalence estimates among pregnant women attending antenatal care facilities in sub-Saharan Africa.	Cross-sectional prevalence studies	171	Multiple	Yes
Annals of Internal Medicine	Nelson [7]	Observational Epidemiology	To determine what factors increase risk for breast cancer in women aged 40 to 49 years and the magnitude of risk for each factor.	RCTs, Obs Studies, SRs, MAs	95	Multiple	Yes
Annals of Internal Medicine	Imperiale [8]	Observational Epidemiology	To determine the validity of studies about The risk for colorectal cancer (CRC) is unclear for persons who have first-degree relatives with adenomatous polyps (adenomas).	cross-sectional, case– control, and cohort study designs	12	Single	No
British Journal of General Practice	Gardiner [9]	Qualitative	To explore factors that support artnership working between specialist and generalist palliative care providers.	Qualitative, quantitative and mixed methods and one case study	22	(Multiple)	Yes
Social Science & Medicine	Dugas [10]	Other Health Intervention	To conduct a systematic review and meta-analysis of randomized controlled trials evaluating the efficacy of different decision aid tools compared to regular care for women facing several options in the specific field of obstetric care.	RCTs	10	Multiple	Yes
Social Science & Medicine	Hilber [11]	Qualitative	To systematically review qualitative research about vaginal practices in Sub-Saharan Africa, which used meta-ethnographic methods to understand their origins, their meanings for the women who use them, and how they have evolved in time and place.	Qualitative	16	(Multiple)	Yes

PLoS Medicine	Oram [12]	Observational Epidemiology	To estimate: (1) The prevalence of violence whilst trafficked and the risk of violence among trafficked people; (2) The prevalence of physical, mental, and sexual health problems, including HIV/AIDS, among trafficked people; and the risk of these problems among trafficked people; (3) The pooled prevalence of violence and of physical, mental, and sexual health problems, including HIV/AIDS, among trafficked people; and the pooled risk of these problems among trafficked people.	Prevalence studies	19	Multiple	Yes
American Journal of Public Health	Rueda [13]	Other Health Intervention	To systematically reviewed the literature on the impact of returning to work on health among working-aged adults.	Controlled studies (e.g., randomized controlled trials and quasi-experimental studies), cohort studies, and case-control studies	18	Multiple	No
American Journal of Public Health	Varda [14]	Qualitative	To explore and analyse how findings from public affairs research can inform public health research and practice, specifically in the area of interorganizational collaboration, one of the most promising practice-based approaches in the public health field.	Case analysis, Literature review, Meta-analysis, Mixed methods, Network analysis, Opinion, Qualitative, Quantitative, Theory building	151	(Multiple)	Yes

Preventive Medicine	Escalante [15]	Other Health Intervention	To assess the effectiveness of different physical exercise interventions on the lipid profile (high-density lipoprotein cholesterol (HDL-C), low-density lipoprotein cholesterol (LDL-C), total cholesterol (TC), and triglycerides (TG)) of obese children.	RCTs, CCT	7	Multiple	Yes
BMJ	Hemmingsen [16]	Health Technology Intervention	To compare the benefits and harms of metformin and insulin versus insulin alone as reported in randomised clinical trials of patients with type 2 diabetes.	RCTs	26	Multiple	Yes
BMJ	Hu [17]	Observational Epidemiology	To summarise evidence on the association between white rice consumption and risk of type 2 diabetes and to quantify the potential dose-response relation	Cohorts	7	Single	Yes
BMJ	Asbridge [18]	Observational Epidemiology	To determine whether the acute consumption of cannabis (cannabinoids) by drivers increases the risk of a motor vehicle collision.	case control and culpablity studies	9	Single	Yes
BMJ	Karagiannis [19]	Health Technology Intervention	To assess the efficacy and safety of dipeptidyl peptidase-4 (DPP-4) inhibitors compared with metformin as monotherapy, or with other commonly used hypoglycaemic drugs combined with metformin, in adults with type 2 diabetes mellitus.	RCTs	19	Multiple	Yes

BMJ	Orrow [20]	Other Health Intervention	To determine whether trials of physical activity promotion based in primary care show sustained effects on physical activity or fitness in sedentary adults, and whether exercise referral interventions are more effective than other interventions.	RCTs	15	Multiple	Yes
Lancet	Clark [21]	Observational Epidemiology	To establish whether a difference in SBP between arms is associated with ipsilateral angio graphically proven subclavian stenosis on the side of the arm with the lowest pressure, with peripheral or cardiovascular disease, and with an increased risk of cardiovascular-related or all-cause mortality.	Cohort or cross-sectional studies	28	Multiple	Yes
Lancet	Hughes [22]	Observational Epidemiology	To identify the characteristics and coverage of research for the prevalence and risk of violence against adults with disabilities; assess the quality of this research; and synthesise evidence on the prevalence and risk of violence against adults with disabilities to identify knowledge gaps and research priorities.	cross-sectional, case control, or cohort (including longitudinal) study	26	Multiple	Yes
JAMA	Jackson [23]	Health Technology Intervention	To assess botulinum toxin A for the prophylactic treatment of headaches in adults	RCTs	31	Multiple	Yes
Annals of Internal Medicine	Palmer [24]	Health Technology Intervention	To summarise the effects of antiplatelet treatment on cardiovascular events, mortality, and bleeding in persons with CKD.	RCTs	40	Multiple	Yes

Annals of Internal Medicine	Hsu [25]	Observational Epidemiology	To systematically review observational studies for benefits and harms of oseltamivir, zanamivir, amantadine, or rimantadine in the treatment of influenza	Observational Studies	74	Multiple	Yes
Annals of Internal Medicine	Chartrand [26]	Health Technology Intervention	To examine the accuracy of rapid influenza diagnostic tests (RIDTs) in adults and children with influenza-like illness and evaluate factors associated with higher accuracy.	Studies that compared RIDTs with a reference standard of either reverse transcriptase PCR (first choice) or viral culture. Excluded case-control.	159	Single	Yes
Annals of Internal Medicine	Fink [27]	Other Health Intervention	To summarise evidence about the benefits and harms of screening for and monitoring and treatment of CKD stages 1 to 3 in adults.	RCTs	110	Multiple	Yes
Annals of Internal Medicine	Neumann [28]	Health Technology Intervention	To evaluate the benefits and harms of oral direct factor Xa inhibitors versus low-molecular-weight heparin (LMWH) in patients undergoing total hip or knee replacement.	RCTs	25	Multiple	Yes
Annals of Internal Medicine	Sobieraj [29]	Health Technology Intervention	To compare the benefits and harms of prolonged versus standard-duration thromboprophylaxis after major orthopedic surgery in adults	RCTs	8	Multiple	Yes
PLoS Medicine	Boussageon [30]	Health Technology Intervention	To review all available evidence to evaluate the risk-to-benefit balance of metformin in T2DM patients based on cardiovascular morbidity and mortality using a systematic review and meta-analysis of controlled trials.	RCTs	13	Multiple	Yes

PLoS Medicine	Herbert [31]	Observational Epidemiology	To describe the proportion of caregivers who seek medical care once they recognize their neonate is ill or is suspected to be ill in LMICs.	Trials, Surveys	22	Single	No
Social Science & Medicine	Meijer [32]	Observational Epidemiology	To conduct a systematic literature review of previous studies and to quantify the association between area-level socioeconomic status (ALSES) and all-cause mortality in a meta-analysis.	Observational Studies	40	Single	Yes
Social Science & Medicine	Rietjens [33]	Observational Epidemiology	To detect whether there are differences in the prevalence of medical end-oflife decisions in 'vulnerable' patient groups.	Retrospective or Prospective cohorts	51	Multiple	Yes
British Journal of General Practice	France [34]	Observational Epidemiology	To identify prospective cohort studies of multimorbidity in primary care to determine: their nature, scope and key findings; themethodologies used; and gaps in knowledge.	Prospective, longitudinal design	6	Multiple	No
Addiction	Jackson [35]	Other Health Intervention	To identify and assess the effectiveness of experimental studies of interventions that report on multiple risk behaviour outcomes in young people	RCT, CCT	18	Multiple	No
Addiction	Hedrich [36]	Health Technology Intervention	To review evidence on the effectiveness of opioid maintenance treatment (OMT) in prison and post-release	RCTs, Observational studies	21	Multiple	No

Annals of Family Medicine	Huntley [37]	Observational Epidemiology	To identify measures of multimorbidity and morbidity burden suitable for use in research in primary care and community populations, and to investigate their validity in relation to anticipated associations with patient characteristics, process measures, and health outcomes.	Quantitative studies of any design	194	Multiple	No
Annals of Family Medicine	Fortin [38]	Observational Epidemiology	To identify and compare studies reporting the prevalence of multimorbidity and to suggest methodologic aspects to be considered in the conduct of such studies.	Prevalence studies	21	Single	No
Annals of Family Medicine	Hayward [39]	Health Technology Intervention	To systematically review and meta-analyse the effects of intranasal corticosteroids on the symptoms of acute sinusitis.	RCTs	6	Multiple	Yes
Pediatrics	Mohamed [40]	Observational Epidemiology	To systematically review and meta-analyze the association between transfusion and NEC (TANEC), identify predictors of TANEC, and the assess impact of TANEC on outcomes.	Observational Studies	12	Multiple	Yes
Pediatrics	Seida [41]	Health Technology Intervention	To systematically review the effectiveness and safety of first- (FGA) and second-generation antipsychotics (SGA) for patients aged #24 years with psychiatric and behavioral conditions.	RCTs, Cohorts	81	Multiple	Yes

Pediatrics	van Noort-van der Spek [42]	Observational Epidemiology	To investigate the developmental course of language functions in preterm-born children compared with term-born children throughout childhood by performing ameta-analysis.	Case-control studies	17	Multiple	Yes
Pediatrics	Bruijning-Verhagen [43]	Observational Epidemiology	To summarise the existing evidence and produce reliable estimates of nRV incidence, in pediatric settings in Europe and North America.	Observational Studies	20	Single	Yes
Pediatrics	Burke [44]	Observational Epidemiology	To provide estimates of the prospective effect of smoking by parents or household members on the risk of wheeze and asthma at different stages of childhood.	Cohort studies	79	Multiple	Yes
Pediatrics	Kidger [45]	Other Health Intervention	To synthesise the evidence for the effect on adolescent emotional health of (1) interventions targeting the school environment and (2) the school environment in cohort studies.	Controlled intervention studies and cohort studies	28	Multiple	No
Cochrane	Alfirevic [46]	Health Technology Intervention	To assess whether the use of cervical stitch in singleton pregnancy at high risk of pregnancy loss based on a woman's history and/or ultrasound finding of 'short cervix' and/or physical exam improves subsequent obstetric care and fetal outcome.	RCTs	12	Multiple	Yes
Cochrane	Callaghan [47]	Health Technology Intervention	To assess the effects, primarily on pain and function, of patellar taping for treating patellofemoral pain syndrome in adults.	RCTs, Quasi-RCTs	5	Multiple	Yes

Cochrane	Van Miert [48]	Health Technology Intervention	To assess the efficacy and safety of different interventions used to restore patency of occluded CVC lumens, in adults and children.	RCTs	7	Multiple	Yes
Cochrane	Novikova [49]	Health Technology Intervention	To establish the efficacy and safety of local anaesthetic nerve blocks for pain relief in labour.	RCTs	12	Multiple	Yes
Cochrane	Croft [50]	Health Technology Intervention	To assess the safety and effectiveness of helminth therapy in people with allergic rhinitis.	RCTs	2	Multiple	No
Cochrane	Lee [51]	Health Technology Intervention	To investigate the correct positioning (or mobilisation) and examine the effects of spinal bracing to relieve pain or to prevent further vertebral collapse in patients with MSCC.	RCTs	0	Multiple	No
Cochrane	Rolinski [52]	Health Technology Intervention	To assess the efficacy, safety and tolerability of cholinesterase inhibitors in dementia with Lewy bodies (DLB), Parkinson's disease with dementia (PDD), and cognitive impairment in Parkinson's disease falling short of dementia (CIND-PD) (considered as separate phenomena and also grouped together as Lewy body disease).	RCTs	6	Multiple	Yes

Cochrane	Hurwitz [53]	Health Technology Intervention	To determine if treatment with tricyclic antidepressants: 1) improves the core features of autism, including restricted social interaction, restricted communication, and stereotypical and repetitive behaviours; 2) improves non-core features such as challenging behaviours; 3) improves comorbid states, such as depression and anxiety; 4) causes adverse effects.	RCTs	3	Multiple	No
Cochrane	Drahota [54]	Health Technology Intervention	To assess the effect of hospital environments on adult patient health-related outcomes.	Randomised and non-randomised controlled trials, controlled before-and-after studies, and interrupted times series	102	Multiple	Yes
Cochrane	Gordijn [55]	Observational Epidemiology	To examine the occurrence and duration of HPA axis suppression after (each cycle of) glucocorticoid therapy for childhood ALL.	All study designs, except case reports and patient series with fewer than 10 patients,	7	Single	No
Cochrane	Suo [56]	Health Technology Intervention	To assess the efficacy and safety of oral traditional Chinese medicine (TCM) for adhesive small bowel obstruction.	RCTs and Quasi-RCTs	5	Multiple	Yes
Cochrane	Fedorowicz [57]	Health Technology Intervention	To determine the effectiveness and safety of beta2-adrenoceptor agonists in the treatment of primary dysmenorrhoea.	RCTs	5	Multiple	No
Cochrane	Han [58]	Other Health Intervention	To assess the effects of different types of management strategies for pregnant women with hyperglycaemia not meeting diagnostic criteria for GDM and T2DM	RCTs and CRCTs	4	Multiple	Yes

Cochrane	Akl [59]	Observational Epidemiology	To evaluate the effects of using alternative statistical presentations of the same risks and risk reductions on understanding, perception, persuasiveness and behaviour of health professionals, policy makers, and consumers.	Randomized and non-randomized controlled parallel and cross-over studies.	35	Multiple	Yes

RCTs = Randomised-controlled trials; CRCTs = Cluster randomised-controlled trials; CCTs = Controlled clinical trial

Appendix Table 2 – Detailed description of methods for ranking individual study quality

Publication	First Author	Summary assessment for risk of bias allowing ranking given	How Ranking Achieved	Justification for Ranking Approach	Rationale for study assessment
BMJ	Prochaska [2]	Yes	Only one criteria distinguished studies	None	Only one of the criteria was not fulfilled by some studies. No rationale given for ranking otherwise.
BMJ	Ker [4]	Yes	Allocation concealment and blinding	None	Sensitivity analyses based on allocation concealment and blinding of outcomes - so possibly prioritised for ranking. No rationale given.
JAMA	Hempel [5]	Yes	Unclear	None	No
Annals of Internal Medicine	Nelson [7]	Yes	Good study meets all criteria, Poor study has a fatal flaw.		Yes. Guidance doc: In general, a good study meets all criteria for that study design; a fair study does not meet all criteria but is judged to have no fatal flaw that invalidates its results; and a poor study contains a fatal flaw.
Social Science & Medicine	Dugas [10]	Yes	Appears to prioritise on basis of two criteria	None	No. As only mentioned two criteria, appears that based on prioritising these two domains.

PLoS Medicine	Oram [12]	Yes	Two sub-domains presented as more important	None	Scores for two sub-domains—the quality of studies' sampling strategies and the quality of measurements—are presented alongside the total quality score. Scores for other sub-domains are not shown.
Preventive Medicine	Escalante [15]	Yes	Scored over 50% criteria	None	Table 2 desrcibes individual criteria. Text focuses on studies that scored over 50% of criteria: Only two of the seven studies (Ferguson et al., 1999; Farpour-Lambert et al., 2009) satisfied at least 50% of the quality criteria (four or more quality criteria) (Table 2). Confusingly, the table lists 9 criteria, not 8.
BMJ	Hemmingsen [16]	Yes	Sequence generation, allocation concealment, blinding.	None	"Only two trials6 44 described adequate sequence generation, allocation concealment, and blinding of participants and investigators, which our protocol had prespecified as trials with lower risk of bias.15"
BMJ	Hu [17]	Yes	Total quality score over 12	None	Total score presented - over 12 treated as high scoring
BMJ	Karagiannis [19]	Yes	High risk of bias if high risk in any domain, Low if all key domains of low bias, Unclear in other cases	Key domains stated as "all domains except random sequence generation and allocation concealment" but no justification	We summarised the risk of bias of all six domains to produce an overall risk of bias for every outcome within every different report. This was deemed high in the presence of high bias in any domain, low if all key domains (all domains except random sequence generation and allocation concealment) were of low bias, and unclear in all other cases.
Lancet	Hughes [22]	Yes	Total quality score	None	No domain prioritised. Adds up individual criteria to give

					overall score
JAMA	Jackson [23]	Yes	Ranking possible by Jadad score but component approach used for each domain.	Each domain's potential effect on results evaluated.	Ranking possible by Jadad score. Quality assessment was performed by using a component approach in which each domain's potential effect on our results was evaluated. In addition,wetested the effect of study sponsorship and use of intention-to-treat analysis (for trials with losses to follow-up). We assessed for small study effects (publication bias) using the methods of Peters et al for dichotomous and Egger et al for continuous outcomes. We explored potential sources of heterogeneity using stratified analysis and meta-regression.
Annals of Internal Medicine	Fink [27]	Yes	None	None	None given
Annals of Internal Medicine	Sobieraj [29]	Yes	Good study needs to meet all criteria		Not clear how the overall score was derived although table 2 does provide brief ad hoc explanation for why studies rated as 'fair' rather than good. The text suggests 'good' studies needed to meet all 11 criteria.
PLoS Medicine	Boussageon [30]	Yes	Total Jadad score and double blinding	None	Table 1 suggests Jadad score and double-blinding important
Social Science & Medicine	Meijer [32]	Yes	Majority of criteria	None	Appears to use a majority to determine whether study classified as high, med or low quality

Addiction	Jackson [35]	Yes	Equal weight to each domain. Strong - no weak ratings, Mod - 1 weak rating, Weak - 2+ weak ratings	None	Tool guidance states: Strong -no weak ratings, Mod - 1 weak rating, Weak - 2+ weak ratings
Annals of Family Medicine	Fortin [38]	Yes	Total score from quality criteria	None	Number of criteria totalled
Annals of Family Medicine	Hayward [39]	Yes	Unclear	None	No. Table 2 summarises ind criteria ax for each study - text says two of the studies are of low methodological quality but no reasons provided. All 6 included studies demonstrated adequate allocation, concealment, blinding, percentage participation, and comparability of groups both at baseline and in provision of care apart from the intervention; however, 3 studies did not report the method of randomization (Table 2). We therefore performed a sensitivity analysis excluding these studies.
Pediatrics	Mohamed [40]	Yes	Weighted score (with domains allocated one or two points)	None	Score based on summing except two domains marked out of 2 (case and control comparability, ascertainment of exposure)
Pediatrics	Seida [41]	Yes	Unclear	None	Not clear
Pediatrics	van Noort-van der Spek [42]	Yes	Overall score	None	Overall score used

Pediatrics	Bruijning-Verhagen [43]	Yes	Unclear	None	Low or high bias in Table 1. No rationale given
Pediatrics	Burke [44]	Yes	Overall score	None	Newcastle-Ottawa Scale
Cochrane	Alfirevic [46]	Yes	Sequence generation and allocation concealment prioritised	None	We planned to perform sensitivity analysis on the primary outcomes based on trial quality, separating high-quality trials from trials of lower quality. For the purposes of this sensitivity analysis, we defined high quality as a trials rated as 'low risk of bias' for sequence generation and allocation concealment.
Cochrane	Fedorowicz [57]	Yes	Low risk of bias requires all criteria to be met	None	Not by prioritising domain. "The overall risk of bias of each of the included studies has also been reported according to the following categories.<br>• Low risk of bias (plausible bias unlikely to seriously alter the results) if all criteria were met.<br>• Unclear risk of bias (plausible bias that raises some doubt about the results) if one or more criteria were assessed as unclear.<br>• High risk of bias (plausible bias that seriously weakens confidence in the results) if one or more criteria were not met.
Cochrane	Han [58]	Yes	Unclear	None	Three of the four included studies were at moderate to high risk of bias and one study (Grant 2011) was at low to moderate risk of bias. Magnitude and direction of bias rationale not stated.

Cochrane	Akl [59]	Yes	At least two of the four criteria required	None	“We also conducted pre-planned sensitivity analyses excluding studies:<br>• of lower methodological quality (ie those which did not meet at least two of the four methodological criteria);”

Appendix Box

List of other methods used for incorporating quality in synthesis for systematic reviews

- Univariate analysis on basis of sample size and quality assessment score [22]
- Assessment of strength of evidence on basis of Agency for Healthcare Research and Quality and the Effective Health-care Program guidelines [27, 60]
- Likely direction and influence of bias as assessed by the reviewers [36]
- Studies controlling for confounders used preferentially in meta-analysis [40]

Appendix references

1 Herbert K, Plugge E, Foster C*, et al.* Prevalence of risk factors for non-communicable diseases in prison populations worldwide: a systematic review. *The Lancet* 2012;**379**:1975-82.

2 Prochaska JJ, Hilton JF. Risk of cardiovascular serious adverse events associated with varenicline use for tobacco cessation: systematic review and meta-analysis. *BMJ* 2012;**344**.

3 Thangaratinam S, Rogozińska E, Jolly K*, et al.* Effects of interventions in pregnancy on maternal weight and obstetric outcomes: meta-analysis of randomised evidence. *BMJ* 2012;**344**.

4 Ker K, Edwards P, Perel P*, et al.* Effect of tranexamic acid on surgical bleeding: systematic review and cumulative meta-analysis. *BMJ* 2012;**344**.

5 Hempel S NSJMAR, et al. Probiotics for the prevention and treatment of antibiotic-associated diarrhea: A systematic review and meta-analysis. *JAMA: The Journal of the American Medical Association* 2012;**307**:1959-69.

6 Chico R MPACMDRCCD. Prevalence of malaria and sexually transmitted and reproductive tract infections in pregnancy in sub-saharan africa: A systematic review. *JAMA: The Journal of the American Medical Association* 2012;**307**:2079-86.

7 Nelson HD, Zakher B, Cantor A*, et al.* Risk Factors for Breast Cancer for Women Aged 40 to 49 YearsA Systematic Review and Meta-analysis. *Annals of Internal Medicine* 2012;**156**:635-48.

8 Imperiale TF, Ransohoff DF. Risk for Colorectal Cancer in Persons With a Family History of Adenomatous PolypsA Systematic Review. *Annals of Internal Medicine* 2012;**156**:703-9.

9 Gardiner C, Gott M, Ingleton C. Factors supporting good partnership working between generalist and specialist palliative care services: a systematic review. *British Journal of General Practice* 2012;**62**:e353-e62.

10 Dugas M, Shorten A, Dubé E*, et al.* Decision aid tools to support women's decision making in pregnancy and birth: A systematic review and meta-analysis. *Social Science & Medicine* 2012;**74**:1968-78.

11 Martin Hilber A, Kenter E, Redmond S*, et al.* Vaginal practices as women's agency in Sub-Saharan Africa: A synthesis of meaning and motivation through meta-ethnography. *Social Science & Medicine* 2012;**74**:1311-23.

12 Oram S, Stöckl H, Busza J*, et al.* Prevalence and Risk of Violence and the Physical, Mental, and Sexual Health Problems Associated with Human Trafficking: Systematic Review. *PLoS Med* 2012;**9**:e1001224.

13 Rueda S, Chambers L, Wilson M*, et al.* Association of Returning to Work With Better Health in Working-Aged Adults: A Systematic Review. *American Journal of Public Health* 2012;**102**:541-56.

14 Varda D, Shoup JA, Miller S. A Systematic Review of Collaboration and Network Research in the Public Affairs Literature: Implications for Public Health Practice and Research. *American Journal of Public Health* 2011;**102**:564-71.

15 Escalante Y, Saavedra JM, García-Hermoso A*, et al.* Improvement of the lipid profile with exercise in obese children: A systematic review. *Preventive Medicine* 2012;**54**:293-301.

16 Hemmingsen B, Christensen LL, Wetterslev J*, et al.* Comparison of metformin and insulin versus insulin alone for type 2 diabetes: systematic review of randomised clinical trials with meta-analyses and trial sequential analyses. *BMJ* 2012;**344**.

17 Hu EA, Pan A, Malik V*, et al.* White rice consumption and risk of type 2 diabetes: meta-analysis and systematic review. *BMJ* 2012;**344**.

18 Asbridge M, Hayden JA, Cartwright JL. Acute cannabis consumption and motor vehicle collision risk: systematic review of observational studies and meta-analysis. *BMJ* 2012;**344**.

19 Karagiannis T, Paschos P, Paletas K*, et al.* Dipeptidyl peptidase-4 inhibitors for treatment of type 2 diabetes mellitus in the clinical setting: systematic review and meta-analysis. *BMJ* 2012;**344**.

20 Orrow G, Kinmonth A-L, Sanderson S*, et al.* Effectiveness of physical activity promotion based in primary care: systematic review and meta-analysis of randomised controlled trials. *BMJ* 2012;**344**.

21 Clark CE, Taylor RS, Shore AC*, et al.* Association of a difference in systolic blood pressure between arms with vascular disease and mortality: a systematic review and meta-analysis. *The Lancet* 2012;**379**:905-14.

22 Hughes K, Bellis MA, Jones L*, et al.* Prevalence and risk of violence against adults with disabilities: a systematic review and meta-analysis of observational studies. *The Lancet* 2012;**379**:1621-9.

23 Jackson Jl KAHY. Botulinum toxin a for prophylactic treatment of migraine and tension headaches in adults: A meta-analysis. *JAMA: The Journal of the American Medical Association* 2012;**307**:1736-45.

24 Palmer SC, Di Micco L, Razavian M*, et al.* Effects of Antiplatelet Therapy on Mortality and Cardiovascular and Bleeding Outcomes in Persons With Chronic Kidney DiseaseA Systematic Review and Meta-analysis. *Annals of Internal Medicine* 2012;**156**:445-59.

25 Hsu J, Santesso N, Mustafa R*, et al.* Antivirals for Treatment of Influenza: A Systematic Review and Meta-analysis of Observational Studies. *Annals of Internal Medicine* 2012;**156**:512-24.

26 Chartrand C, Leeflang MMG, Minion J*, et al.* Accuracy of Rapid Influenza Diagnostic Tests: A Meta-analysis. *Annals of Internal Medicine* 2012;**156**:500-11.

27 Fink HA, Ishani A, Taylor BC*, et al.* Screening for, Monitoring, and Treatment of Chronic Kidney Disease Stages 1 to 3: A Systematic Review for the U.S. Preventive Services Task Force and for an American College of Physicians Clinical Practice Guideline. *Annals of Internal Medicine* 2012;**156**:570-81.

28 Neumann I, Rada G, Claro JC*, et al.* Oral Direct Factor Xa Inhibitors Versus Low-Molecular-Weight Heparin to Prevent Venous Thromboembolism in Patients Undergoing Total Hip or Knee Replacement: A Systematic Review and Meta-analysis. *Annals of Internal Medicine* 2012;**156**:710-9.

29 Sobieraj DM, Lee S, Coleman CI*, et al.* Prolonged Versus Standard-Duration Venous Thromboprophylaxis in Major Orthopedic Surgery: A Systematic Review. *Annals of Internal Medicine* 2012;**156**:720-7.

30 Boussageon R, Supper I, Bejan-Angoulvant T*, et al.* Reappraisal of Metformin Efficacy in the Treatment of Type 2 Diabetes: A Meta-Analysis of Randomised Controlled Trials. *PLoS Med* 2012;**9**:e1001204.

31 Herbert HK, Lee ACC, Chandran A*, et al.* Care Seeking for Neonatal Illness in Low- and Middle-Income Countries: A Systematic Review. *PLoS Med* 2012;**9**:e1001183.

32 Meijer M, Röhl J, Bloomfield K*, et al.* Do neighborhoods affect individual mortality? A systematic review and meta-analysis of multilevel studies. *Social Science & Medicine* 2012;**74**:1204-12.

33 Rietjens JAC, Deschepper R, Pasman R*, et al.* Medical end-of-life decisions: Does its use differ in vulnerable patient groups? A systematic review and meta-analysis. *Social Science & Medicine* 2012;**74**:1282-7.

34 France EF, Wyke S, Gunn JM*, et al.* Multimorbidity in primary care: a systematic review of prospective cohort studies. *British Journal of General Practice* 2012;**62**:e297-e307.

35 Jackson C, Geddes R, Haw S*, et al.* Interventions to prevent substance use and risky sexual behaviour in young people: a systematic review. *Addiction* 2012;**107**:733-47.

36 Hedrich D, Alves P, Farrell M*, et al.* The effectiveness of opioid maintenance treatment in prison settings: a systematic review. *Addiction* 2012;**107**:501-17.

37 Huntley AL, Johnson R, Purdy S*, et al.* Measures of Multimorbidity and Morbidity Burden for Use in Primary Care and Community Settings: A Systematic Review and Guide. *The Annals of Family Medicine* 2012;**10**:134-41.

38 Fortin M, Stewart M, Poitras M-E*, et al.* A Systematic Review of Prevalence Studies on Multimorbidity: Toward a More Uniform Methodology. *The Annals of Family Medicine* 2012;**10**:142-51.

39 Hayward G, Heneghan C, Perera R*, et al.* Intranasal Corticosteroids in Management of Acute Sinusitis: A Systematic Review and Meta-Analysis. *The Annals of Family Medicine* 2012;**10**:241-9.

40 Mohamed A, Shah PS. Transfusion Associated Necrotizing Enterocolitis: A Meta-analysis of Observational Data. *Pediatrics* 2012;**129**:529-40.

41 Seida JC, Schouten JR, Boylan K*, et al.* Antipsychotics for Children and Young Adults: A Comparative Effectiveness Review. *Pediatrics* 2012;**129**:e771-e84.

42 van Noort-van der Spek IL, Franken M-CJP, Weisglas-Kuperus N. Language Functions in Preterm-Born Children: A Systematic Review and Meta-analysis. *Pediatrics* 2012;**129**:745-54.

43 Bruijning-Verhagen P, Quach C, Bonten M. Nosocomial Rotavirus Infections: A Meta-analysis. *Pediatrics* 2012;**129**:e1011-e9.

44 Burke H, Leonardi-Bee J, Hashim A*, et al.* Prenatal and Passive Smoke Exposure and Incidence of Asthma and Wheeze: Systematic Review and Meta-analysis. *Pediatrics* 2012;**129**:735-44.

45 Kidger J, Araya R, Donovan J*, et al.* The Effect of the School Environment on the Emotional Health of Adolescents: A Systematic Review. *Pediatrics* 2012;**129**:925-49.

46 Alfirevic Z, Stampalija T, Roberts D*, et al.* Cervical stitch (cerclage) for preventing preterm birth in singleton pregnancy. *Cochrane Database of Systematic Reviews*: John Wiley & Sons, Ltd 2012.

47 Callaghan Michael J, Selfe J. Patellar taping for patellofemoral pain syndrome in adults. *Cochrane Database of Systematic Reviews*: John Wiley & Sons, Ltd 2012.

48 van Miert C, Hill R, Jones L. Interventions for restoring patency of occluded central venous catheter lumens. *Cochrane Database of Systematic Reviews*: John Wiley & Sons, Ltd 2012.

49 Novikova N, Cluver C. Local anaesthetic nerve block for pain management in labour. *Cochrane Database of Systematic Reviews*: John Wiley & Sons, Ltd 2012.

50 Croft Ashley M, Bager P, Kumar S. Helminth therapy (worms) for allergic rhinitis. *Cochrane Database of Systematic Reviews*: John Wiley & Sons, Ltd 2012.

51 Lee Siew H, Cox Katherine M, Grant R*, et al.* Patient positioning (mobilisation) and bracing for pain relief and spinal stability in metastatic spinal cord compression in adults. *Cochrane Database of Systematic Reviews*: John Wiley & Sons, Ltd 2012.

52 Rolinski M, Fox C, Maidment I*, et al.* Cholinesterase inhibitors for dementia with Lewy bodies, Parkinson's disease dementia and cognitive impairment in Parkinson's disease. *Cochrane Database of Systematic Reviews*: John Wiley & Sons, Ltd 2012.

53 Hurwitz R, Blackmore R, Hazell P*, et al.* Tricyclic antidepressants for autism spectrum disorders (ASD) in children and adolescents. *Cochrane Database of Systematic Reviews*: John Wiley & Sons, Ltd 2012.

54 Drahota A, Ward D, Mackenzie H*, et al.* Sensory environment on health-related outcomes of hospital patients. *Cochrane Database of Systematic Reviews*: John Wiley & Sons, Ltd 2012.

55 Gordijn Maartje S, Gemke Reinoud JBJ, van Dalen Elvira C*, et al.* Hypothalamic-pituitary-adrenal (HPA) axis suppression after treatment with glucocorticoid therapy for childhood acute lymphoblastic leukaemia. *Cochrane Database of Systematic Reviews*: John Wiley & Sons, Ltd 2012.

56 Suo T, Gu X, Andersson R*, et al.* Oral traditional Chinese medication for adhesive small bowel obstruction. *Cochrane Database of Systematic Reviews*: John Wiley & Sons, Ltd 2012.

57 Fedorowicz Z, Nasser M, Jagannath Vanitha A*, et al.* Beta2-adrenoceptor agonists for dysmenorrhoea. *Cochrane Database of Systematic Reviews*: John Wiley & Sons, Ltd 2012.

58 Han S, Crowther Caroline A, Middleton P. Interventions for pregnant women with hyperglycaemia not meeting gestational diabetes and type 2 diabetes diagnostic criteria. *Cochrane Database of Systematic Reviews*: John Wiley & Sons, Ltd 2012.

59 Akl Elie A, Oxman Andrew D, Herrin J*, et al.* Using alternative statistical formats for presenting risks and risk reductions. *Cochrane Database of Systematic Reviews*: John Wiley & Sons, Ltd 2011.

60 Owens DK, Lohr KN, Atkins D*, et al.* AHRQ Series Paper 5: Grading the strength of a body of evidence when comparing medical interventions—Agency for Healthcare Research and Quality and the Effective Health-Care Program. *J Clin Epidemiol* 2010;**63**:513-23.
